# Regional inequalities in heart failure hospitalisation and in-hospital mortality in Spain (2016–2022): a nationwide, observational study

**DOI:** 10.1016/j.lanepe.2025.101549

**Published:** 2025-12-02

**Authors:** Carolina Ortiz-Cortés, Leticia Fernández-Friera, José M. Gámez, Ángel Cequier Fillat, Clara Bonanad Lozano, Antonia Sambola, Lorenzo Fácila Rubio, Luis Rodríguez Padial, Raquel Campuzano Ruíz, Milagros Pedreira Pérez, José M. de la Torre Hernández, Nicolás Rosillo, Sofía Sánchez Fernández, Náyade del Prado, José Luis Bernal, Cristina Fernández Pérez, Diego Alvaredo, Sergio Rico-Martín, Francisco Javier Elola-Somoza

**Affiliations:** a Department of Cardiology, Hospital Universitario Fundación Alcorcón, Madrid, Spain; b HM CIEC Madrid (Centro Integral de Enfermedades Cardiovasculares), Hospital Universitario HM Montepríncipe, HM Hospitales, HM Faculty of Health Sciences, Camilo José Cela University, HM Hospitales Research Institute, Madrid, Spain; c AtriaClinic, Madrid, Spain; d Department of Cardiology, Hospital Universitario Son Llàtzer, Palma de Mallorca, Spain; e Department of Medicine, University of the Balearic Islands, Palma, Spain; f CIBEROBN (CB12/03/30038), Instituto de Salud Carlos III, Madrid, Spain; g Bellvitge Biomedical Research Institute (IDIBELL), Barcelona, Spain; h Department of Cardiology, Hospital Clínico Universitario de Valencia, Valencia, Spain; i Faculty of Medicine and Dentistry, University of Valencia, Valencia, Spain; j INCLIVA Health Research Institute, Valencia, Spain; k Biomedical Research Networking Centre on Cardiovascular Diseases (CIBERCV), Madrid, Spain; l Department of Cardiology, Hospital Universitari Vall d’Hebron; Vall d’Hebron Research Institute (VHIR), Barcelona, Spain; m CIBERCV, Barcelona, Spain; n Department of Cardiology, Hospital General Universitario de Valencia, Universitat de València, Valencia, Spain; o Department of Cardiology, Hospital Universitario de Toledo, Castilla-La Mancha, Spain; p Department of Cardiology, Complexo Hospitalario Universitario de Santiago de Compostela, Santiago de Compostela, Galicia, Spain; q Department of Cardiology, Hospital Universitario Marqués de Valdecilla, IDIVAL, Santander, Spain; r Fundación Instituto para la Mejora de la Asistencia Sanitaria (IMAS), Cátedra IMAS-Universidad Rey Juan Carlos, Madrid, Spain; s Department of Preventive Medicine, Health Research Institute of Santiago (IDIS), Santiago de Compostela–Barbanza Health Area, University of Santiago de Compostela, Santiago de Compostela, Spain; t Department of Nursing, Nursing and Occupational Therapy College, University of Extremadura, Cáceres, Spain

**Keywords:** Heart failure, Hospitalisation, In-hospital mortality, Regional disparities, Socioeconomic factors, Health determinants

## Abstract

**Background:**

Heart failure remains one of the leading causes of hospital admissions and mortality worldwide, with substantial regional disparities in clinical outcomes. These variations have been linked to differences in socioeconomic conditions, health determinants, and health-care system organisation. The aim of this study was to analyse regional differences in hospitalisation rates and in-hospital mortality for heart failure across Spain’s autonomous communities, and to examine their associations with hospital characteristics and regional health determinants.

**Methods:**

We conducted a nationwide, retrospective, population-based study using Spain’s national hospital discharge database (Conjunto Mínimo Básico de Datos, CMBD) to identify adults (≥18 years) discharged from public general hospitals within the Spanish National Health System (SNS) with a primary diagnosis of heart failure from January 1, 2016, to December 31, 2022. Differences in hospitalisation rates and risk-standardised in-hospital mortality between autonomous communities were analysed, along with associations with hospital characteristics and regional health determinants.

**Findings:**

A total of 764,083 hospitalisations for heart failure were analysed. The mean age of participants was 80.8 ± 10.6 years. The age- and sex-standardised hospitalisation rate was 255 per 100,000 inhabitants, with significant differences between autonomous communities. Crude in-hospital mortality rate was 11.3% (86,426 episodes), with substantial regional variation in risk-standardised mortality rate (ranged from 7.7% to 16.4%) No associations were found between hospital characteristics and risk-standardised mortality rate at the regional level. Regional health determinants were highly collinear; among all determinants assessed, per-capita gross domestic product was independently associated with lower risk-standardised mortality (βGDP_pc_ = −0.77; 95% CI: −1.04 to −0.49; p < 0.001; R2 = 0.77).

**Interpretation:**

Significant regional differences in hospitalisation rates and risk-standardised mortality rates were observed among patients hospitalised for heart failure. Regional disparities in gross domestic product per capita may partly explain these differences. These results have clinical and policy implications, underscoring the need to consider socioeconomic determinants when analysing health outcomes and designing public policies aimed at reducing health inequalities. Our findings might be relevant to other European countries with similar public health systems.

**Funding:**

This work was supported by the 10.13039/100009664Spanish Society of Cardiology (Sociedad Española de Cardiología, SEC).

**Research in context**

**Evidence before this study**

We searched PubMed, Scopus, and the Spanish National Health System Statistical Portal for studies published in English and Spanish from January 1, 2016 to June 30, 2025, using the search terms “heart failure”, “hospitalisation”, “in-hospital mortality”, “regional diferences”, “socioeconomic determinants”, “determinants of health”, “Europe”, “United States”, and “Spain”. We also reviewed national reports from the Instituto Nacional de Estadística (INE), the Spanish Ministry of Health, and previous publications from the RECALCAR project (an initiative of the Spanish Society of Cardiology to analyse the quality of cardiovascular care using administrative health data). Previous studies had documented geographical variability in heart failure outcomes in the “United States” and “Europe”, and one national study in “Spain” (2003–2015) reported differences in heart failure hospitalisations and mortality across autonomous communities. However, no recent nationwide analysis had updated these findings or explored the association with a comprehensive set of health determinants and hospital structural characteristics in the context of a public and decentralised healthcare system.

**Added value of this study**

This study analysed over 760,000 hospital admissions for heart failure in Spain between 2016 and 2022. It provides updated and comprehensive data on regional variation in hospitalisation rates and in-hospital mortality using risk-adjusted models. Importantly, we found no association between hospital structural characteristics and risk-standardised mortality but did identify a significant relationship between regional per capita GDP and mortality outcomes. This is one of the largest studies of its kind in Europe and the first in Spain to integrate administrative hospital data with a wide range of regional health determinants.

**Implications of all the available evidence**

Our findings suggest that socioeconomic factors are key contributors to disparities in hospital outcomes for heart failure across Spain’s autonomous communities. Regional per capita GDP inequalities between regions were associated with differences in regional risk-standardised mortality, and regional per capita GDP was strongly correlated with key social indicators—average disposable household income, educational attainment, unemployment rate, and the prevalence of smoking and obesity, suggesting that regional per capita GDP acts as a composite marker of the regional socioeconomic environment. The absence of association with hospital structural indicators highlights the importance of upstream determinants of health in explaining these differences. These results support the need to incorporate regional socioeconomic context in health policy planning and in the assessment of cardiovascular outcomes, both in Spain and in other countries with public healthcare systems, and should take into consideration when comparing healthcare outcomes among in-country regions and also across European countries.

## Introduction

Heart failure (HF) represents a major global health challenge.^1^ Worldwide, HF incidence has stabilised in recent years^1^^,^^2^; however, its prevalence continues to rise driven by population ageing^1^^,^^3^ and improved survival after cardiovascular diseases that progress to HF. In Europe, contemporary, standardised estimates from the Heart Failure Association indicate a median HF incidence of 3.9 per 1000 person-years and a median prevalence of 1937 per 100,000, with substantial heterogeneity across countries.^4^ Among patients hospitalised for HF, median in-hospital mortality is approximately 8%, and longer-term mortality remains high, with 30–50% of patients dying within 3 years of diagnosis.^1^ Regional differences in HF morbidity and mortality have been described in both the United States and Europe.^5^^,^^6^ These inequalities have been attributed to demographic and epidemiological variations; socioeconomic factors; and differences in the quality, accessibility, utilisation, and effectiveness of health care services.^7^^,^^8^

In this context, regional variation in risk-adjusted hospital outcomes within public, decentralised health systems remains less well characterised. The primary objective of this study was to analyse regional differences in hospitalisation rates and in-hospital mortality for HF across Spain’s autonomous communities. Additionally, we examined the associations between regional hospital structure and selected health determinants and the risk-standardised mortality rate.

## Methods

### Population

This was an observational, retrospective study using data from Spain’s national hospital discharge database (Conjunto Mínimo Básico de Datos, CMBD),^9^ a nationwide, compulsory administrative registry that captures hospital discharge information from all public hospitals in Spain. The database records completed hospitalisation episodes; each episode is closed with an administrative discharge record that includes the discharge disposition (home, transfer, death, etc.). In-hospital mortality was defined as a discharge disposition of death. The population data and indicators of health determinants for each autonomous community were obtained from the National Statistics Institute (INE).^10^ We included patients aged 18 years or older with a primary diagnosis of HF (ICD-10 codes detailed in the Supplementary Material) who were discharged between January 1, 2016, and December 31, 2022, from general hospitals within the Spanish National Health System (Sistema Nacional de Salud, SNS). The SNS provides health care coverage to 96.6% of the Spanish population.

Autonomous communities are distinct geographical regions with political authority over health care budgets, organisations, and the management of public health care. The population of each autonomous community ranges from approximately 300,000 to more than 8.5 million inhabitants, with significant demographic, epidemiological, and socioeconomic differences between them.^11^

To ensure more homogeneous comparisons between autonomous communities, we excluded admissions lasting 1 day or less with discharge to home, admissions with a principal diagnosis within category 14 of the International Classification of Diseases, 10th Revision (ICD-10) (pregnancy, childbirth, and puerperium), and admissions with unknown discharge destinations. Multiple hospitalisations resulting from interhospital transfers were considered single episodes and assigned to the first hospital of contact.

As this study was based exclusively on anonymised population-level data, with no identifiable personal information, informed consent and ethics committee approval were not required.

### Definitions and analysis of outcomes

The outcomes considered were hospitalisation and in-hospital mortality. To describe the overall distribution of events in the study population, we calculated: (1) the hospitalisation rate, defined as the number of hospitalisation episodes divided by the population aged ≥18 years and expressed per 100,000 inhabitants; and (2) the crude in-hospital mortality rate, defined as the number of episodes with discharge due to death divided by the total number of episodes and expressed per 100 episodes. To minimise demographic differences, hospitalisation rates were directly standardised by age and sex for between-region comparisons. To minimise differences in patient age, sex, and comorbidity when comparing mortality across regions, we used the risk-standardised mortality rate derived from the multilevel model.

### Structural hospital variables by autonomous community

The hospital structural variables analysed were four measures at the level of the autonomous community:

1. Percentage of patients admitted to high-complexity hospitals, defined according to the RECALCAR classification—a national scheme that categorises hospitals by service complexity based on the presence of a catheterisation laboratory, the availability of in-house cardiac surgery, and procedure-volume thresholds—with high-complexity hospitals corresponding to Types 3 and 4 (see Supplementary Table S1).
2. Percentage of patients admitted to hospitals with an available coronary care unit (Definition in Supplementary Material).
3. Percentage of patients admitted to hospitals with a cardiac intensive care unit (CICU).
4. Percentage of patients admitted to high-volume hospitals.

### Health determinants

The following health determinants (by autonomous community) were analysed: gross domestic product (GDP) per capita (pc), average disposable household income, population at risk of poverty, income distribution inequality, level of education in the adult population, unemployment rate, percentage of the population who smoke, percentage of the population engaging in high-risk alcohol consumption, obesity, and air pollution from PM 10 to PM 2.5 particulate matter (indicator definitions provided in the Supplementary Material, Supplementary Table S2).

### Statistical analysis

Continuous variables are expressed as means ± standard deviations or medians (interquartile ranges, IQRs), and categorical variables are expressed as absolute numbers and percentages (%). Categorical variables were compared using the χ^2^ test or Fisher's exact test. Continuous variables were compared using Student's t test or the Mann–Whitney test for nonparametric data (for comparisons between two groups) and analysis of variance (ANOVA) with Bonferroni correction or the median test for comparisons between three or more groups. Correlations between variables were analysed using Pearson’s correlation test.

To calculate risk-standardised mortality rate we used the methodology of the Centres for Medicare and Medicaid Services (CMS),^12^ adapting it to the information available in the CMBD, after grouping secondary diagnoses included as adjustment variables according to the condition categories (CCs), which are updated annually by the Agency for Healthcare Research and Quality.^13^ The adjustment variables used in the full risk-adjusted mortality model are detailed in Supplementary Table S3 in the Supplementary Material. We fitted a multilevel logistic regression model with a random intercept at the autonomous-community level, including patients’ age, sex, year of the hospitalisation episode, and comorbidities. Candidate covariates were those listed in Supplementary Table S3, and variable selection was based on minimising the Akaike Information Criterion (AIC).

Structural hospital indicators and health determinants were analysed in ecological models at the autonomous community level for their association with the risk-standardised mortality rate and were not included in the patient-level multilevel model.

On the basis of this model, risk-standardised mortality rate was calculated as the ratio between predicted outcomes (accounting for the specific performance of the autonomous community where the patient was treated) and expected outcomes (reflecting standardised performance on the basis of the mean of all autonomous communities), multiplied by the crude mortality rate. Calibration was assessed graphically by grouping patients into quintiles according to predicted probabilities and plotting the mean predicted probabilities versus the observed probabilities. Discrimination was evaluated using the area under the receiver operating characteristic curve (AUROC). These risk-standardised mortality rate estimates were used to compare in-hospital mortality across autonomous communities and to analyse their associations with structural indicators of hospital management of HF and health determinants. Confidence intervals for risk-standardised mortality rate were estimated using bootstrap techniques.^14^

To calculate the percentage of patients admitted to high-volume hospitals, low- and high-volume hospitals were identified through k-means clustering. The k-means clustering algorithm was applied to achieve maximum intracluster density and minimum intercluster density using two-thirds of the data, with the remaining third used for validation.

Collinearity was assessed eliminating those with variance inflation factors above 10 in regression analyses. Collinearity among health determinants and GDP p.c. was assessed eliminating those with linear correlations (R > 0.50). A linear regression model was used to analyse potential associations at the autonomous community level between structural indicators for HF management in hospitals and health determinants with risk-standardised mortality rate.

Finally, autonomous communities were stratified by GDP p.c. quartiles and linear regression models were developed to assess the association between GDP p.c. autonomous community quartiles with risk-standardised mortality rate for HF.

All the statistical tests were two-sided, and differences were considered statistically significant at p < 0.05. Statistical analyses were performed with Stata 17 and R software (version 4.1.1).

### Role of the funding source

The Spanish Society of Cardiology (SEC) provided financial and institutional support. The study was conceived as an analysis within an SEC strategic programme, and the funder participated in the conception of the study design. The funder had no role in data collection, data analysis, data interpretation, writing of the report, or the decision to submit the manuscript for publication. Authors who accessed and verified the underlying data and the author(s) responsible for the decision to submit are specified in the Contributors section.

## Results

A total of 831,826 hospital admissions for HF were identified during the study period. After exclusions (Supplementary Material, Figure S1), 764,083 episodes were included discharged from 263 hospitals. The mean age was 80.8 ± 10.6 years, and 408,158 (53.4%) of the patients were women. Statistically significant differences were observed between autonomous communities in terms of sex, mean age, and presence of comorbidities (Table 1). The most frequent comorbidities were renal failure (343,204 patients, 44.9%), diabetes mellitus (302,362 patients, 39.6%), and rheumatic or valvular heart disease (263,207, 34.4%).

**Table 1:** Demographics and comorbidity in patients hospitalised for acute heart failure by autonomous community of Spain, 2016–2022.

	Total	Andalucía	Aragón	Asturias	Baleares I	Canarias	Cantabria	Castilla y León	Castilla-La Mancha	Cataluña	C. Valenciana	Extremadura	Galicia	Madrid C.	Murcia R.	Navarra C.F.	País Vasco	Rioja L	p-value
Number of episodes	764,083	102,700	26,970	22,725	11,913	22,937	12,313	33,668	58,940	111,603	82,378	23,423	63,666	116,703	20,792	7676	39,351	6325	
Age (mean ± standard deviation)	80.8 (10.6)	78.3 (11.0)	82.1 (10.0)	81.9 (9.8)	79.0 (11.4)	77.7 (11.5)	81.8 (10.2)	81.7 (10.3)	83.1 (9.8)	80.8 (10.7)	80.2 (10.5)	81.1 (9.9)	81.4 (10.3)	81.7 (10.8)	80.0 (10.2)	81.4 (10.6)	80.5 (10.4)	82.3 (10.0)	<0.001
% women	408,158 (53.4%)	55,580 (54.1%)	13,898 (51.5%)	12,087 (53.2%)	5914 (49.6%)	11,746 (51.2%)	5987 (48.6%)	18,766 (55.7%)	31,319 (53.1%)	59,818 (53.6%)	43,623 (53.0%)	13,218 (56.4%)	32,493 (51.0%)	66,732 (57.2%)	11,138 (53.6%)	3879 (50.5%)	18,708 (47.5%)	3252 (51.4%)	
Metastatic cancer, acute leukaemia and other severe cancers (CC 8–9)	19,632 (2.6%)	2453 (2.4%)	602 (2.2%)	474 (2.1%)	222 (1.9%)	537 (2.3%)	279 (2.3%)	742 (2.2%)	1586 (2.7%)	2869 (2.6%)	2060 (2.5%)	509 (2.2%)	1769 (2.8%)	3761 (3.2%)	486 (2.3%)	151 (2.0%)	1009 (2.6%)	123 (1.9%)	<0.001
Diabetes mellitus (DM) or DM complications except proliferative retinopathy (CC 17–19, 123)	302,362 (39.6%)	46,158 (44.9%)	9581 (35.5%)	8001 (35.2%)	4898 (41.1%)	10,728 (46.8%)	3217 (26.1%)	13,917 (41.3%)	20,400 (34.6%)	45,664 (40.9%)	33,545 (40.7%)	9653 (41.2%)	21,827 (34.3%)	46,001 (39.4%)	9499 (45.7%)	2773 (36.1%)	14,198 (36.1%)	2302 (36.4%)	<0.001
Protein-calorie malnutrition (CC 21)	15,507 (2.0%)	2056 (2.0%)	684 (2.5%)	329 (1.4%)	89 (0.7%)	435 (1.9%)	69 (0.6%)	784 (2.3%)	765 (1.3%)	2402 (2.2%)	972 (1.2%)	287 (1.2%)	580 (0.9%)	5377 (4.6%)	138 (0.7%)	347 (4.5%)	143 (0.4%)	50 (0.8%)	<0.001
Chronic liver disease (CC 27–29)	23,725 (3.1%)	4017 (3.9%)	523 (1.9%)	545 (2.4%)	395 (3.3%)	769 (3.4%)	280 (2.3%)	840 (2.5%)	1253 (2.1%)	4086 (3.7%)	2154 (2.6%)	504 (2.2%)	1749 (2.7%)	4421 (3.8%)	519 (2.5%)	202 (2.6%)	1306 (3.3%)	162 (2.6%)	<0.001
Dementia or other specified brain disorders (CC 51–53)	88,529 (11.6%)	9148 (8.9%)	3147 (11.7%)	2344 (10.3%)	1288 (10.8%)	2003 (8.7%)	968 (7.9%)	4432 (13.2%)	6170 (10.5%)	14,617 (13.1%)	10,207 (12.4%)	2444 (10.4%)	6503 (10.2%)	17,739 (15.2%)	1862 (9.0%)	1211 (15.8%)	3640 (9.3%)	806 (12.7%)	<0.001
Major psychiatric disorders (CC 57–59)	7294 (1.0%)	876 (0.9%)	219 (0.8%)	168 (0.7%)	129 (1.1%)	237 (1.0%)	68 (0.6%)	411 (1.2%)	534 (0.9%)	1279 (1.1%)	701 (0.9%)	269 (1.1%)	521 (0.8%)	1217 (1.0%)	184 (0.9%)	85 (1.1%)	340 (0.9%)	56 (0.9%)	<0.001
Hemiplegia, paraplegia, paralysis, functional disability (CC 70–74, 103–104, 189–190)	5664 (0.7%)	892 (0.9%)	199 (0.7%)	152 (0.7%)	109 (0.9%)	176 (0.8%)	55 (0.4%)	285 (0.8%)	408 (0.7%)	787 (0.7%)	534 (0.6%)	192 (0.8%)	404 (0.6%)	951 (0.8%)	163 (0.8%)	60 (0.8%)	248 (0.6%)	49 (0.8%)	<0.001
Cardiogenic shock	3118 (0.4%)	840 (0.8%)	85 (0.3%)	67 (0.3%)	48 (0.4%)	112 (0.5%)	28 (0.2%)	130 (0.4%)	153 (0.3%)	406 (0.4%)	266 (0.3%)	103 (0.4%)	213 (0.3%)	469 (0.4%)	53 (0.3%)	18 (0.2%)	117 (0.3%)	10 (0.2%)	<0.001
Cardio-respiratory failure (CC 84), plus ICD-10-CM codes R09.01 and R09.02, except cardiogenic shock	226,885 (29.7%)	25,911 (25.2%)	5829 (21.6%)	5589 (24.6%)	3888 (32.6%)	6021 (26.3%)	2019 (16.4%)	14,273 (42.4%)	18,009 (30.6%)	39,177 (35.1%)	16,265 (19.7%)	4458 (19.0%)	22,611 (35.5%)	48,704 (41.7%)	3171 (15.3%)	1598 (20.8%)	7438 (18.9%)	1924 (30.4%)	<0.001
Acute myocardial infarction (CC 86)	5518 (0.7%)	841 (0.8%)	129 (0.5%)	236 (1.0%)	116 (1.0%)	158 (0.7%)	74 (0.6%)	204 (0.6%)	326 (0.6%)	854 (0.8%)	656 (0.8%)	96 (0.4%)	429 (0.7%)	969 (0.8%)	118 (0.6%)	36 (0.5%)	235 (0.6%)	41 (0.6%)	<0.001
Unstable angina and other acute ischaemic heart disease (CC 87)	6147 (0.8%)	1480 (1.4%)	162 (0.6%)	203 (0.9%)	76 (0.6%)	199 (0.9%)	93 (0.8%)	296 (0.9%)	301 (0.5%)	832 (0.7%)	594 (0.7%)	173 (0.7%)	410 (0.6%)	866 (0.7%)	129 (0.6%)	84 (1.1%)	218 (0.6%)	31 (0.5%)	<0.001
Coronary atherosclerosis or angina (CC 88–89)	179,849 (23.5%)	25,056 (24.4%)	5682 (21.1%)	5129 (22.6%)	3083 (25.9%)	5647 (24.6%)	2363 (19.2%)	7205 (21.4%)	12,717 (21.6%)	29,044 (26.0%)	19,937 (24.2%)	5184 (22.1%)	12,492 (19.6%)	28,268 (24.2%)	5220 (25.1%)	1891 (24.6%)	9459 (24.0%)	1472 (23.3%)	<0.001
Valvular and rheumatic heart disease (CC 91)	263,207 (34.4%)	39,817 (38.8%)	8501 (31.5%)	7148 (31.5%)	4154 (34.9%)	7101 (31.0%)	2748 (22.3%)	12,407 (36.9%)	20,117 (34.1%)	39,890 (35.7%)	26,771 (32.5%)	8222 (35.1%)	17,561 (27.6%)	42,245 (36.2%)	6680 (32.1%)	3142 (40.9%)	14,438 (36.7%)	2265 (35.8%)	<0.001
Hypertension (CC 95)	162,896 (21.3%)	20,863 (20.3%)	7029 (26.1%)	3717 (16.4%)	2356 (19.8%)	4115 (17.9%)	1167 (9.5%)	7835 (23.3%)	8700 (14.8%)	25,652 (23.0%)	23,417 (28.4%)	7886 (33.7%)	12,020 (18.9%)	22,327 (19.1%)	3649 (17.6%)	690 (9.0%)	10,378 (26.4%)	1095 (17.3%)	<0.001
Stroke (CC 99–100)	2188 (0.3%)	322 (0.3%)	44 (0.2%)	47 (0.2%)	23 (0.2%)	52 (0.2%)	24 (0.2%)	142 (0.4%)	147 (0.2%)	378 (0.3%)	227 (0.3%)	71 (0.3%)	147 (0.2%)	426 (0.4%)	47 (0.2%)	19 (0.2%)	62 (0.2%)	10 (0.2%)	<0.001
Cerebrovascular disease (CC 101–102, 105)	32,153 (4.2%)	4641 (4.5%)	948 (3.5%)	662 (2.9%)	757 (6.4%)	789 (3.4%)	242 (2.0%)	1655 (4.9%)	2413 (4.1%)	5711 (5.1%)	2783 (3.4%)	1034 (4.4%)	1343 (2.1%)	6250 (5.4%)	1308 (6.3%)	182 (2.4%)	1103 (2.8%)	332 (5.2%)	<0.001
Vascular disease and complications (CC 106–108)	63,168 (8.3%)	9237 (9.0%)	1830 (6.8%)	1513 (6.7%)	912 (7.7%)	1750 (7.6%)	729 (5.9%)	2472 (7.3%)	4038 (6.9%)	11,112 (10.0%)	6103 (7.4%)	1159 (4.9%)	4147 (6.5%)	11,766 (10.1%)	1204 (5.8%)	671 (8.7%)	3906 (9.9%)	619 (9.8%)	<0.001
Chronic obstructive pulmonary disease (COPD) (CC 111)	124,606 (16.3%)	17,534 (17.1%)	4678 (17.3%)	2862 (12.6%)	2270 (19.1%)	3364 (14.7%)	1428 (11.6%)	4953 (14.7%)	8870 (15.0%)	22,617 (20.3%)	12,590 (15.3%)	3333 (14.2%)	8574 (13.5%)	20,674 (17.7%)	2765 (13.3%)	931 (12.1%)	6239 (15.9%)	924 (14.6%)	<0.001
Pneumonia (CC 114–116)	31,237 (4.1%)	5063 (4.9%)	1000 (3.7%)	633 (2.8%)	434 (3.6%)	1280 (5.6%)	247 (2.0%)	1371 (4.1%)	2107 (3.6%)	4390 (3.9%)	2654 (3.2%)	856 (3.7%)	2131 (3.3%)	6626 (5.7%)	817 (3.9%)	327 (4.3%)	1171 (3.0%)	130 (2.1%)	<0.001
Renal failure (CC 135–140)	343,204 (44.9%)	43,288 (42.1%)	12,606 (46.7%)	10,033 (44.1%)	5431 (45.6%)	10,149 (44.2%)	5509 (44.7%)	14,685 (43.6%)	26,414 (44.8%)	55,913 (50.1%)	34,464 (41.8%)	8821 (37.7%)	28,450 (44.7%)	56,683 (48.6%)	8941 (43.0%)	3974 (51.8%)	14,799 (37.6%)	3044 (48.1%)	<0.001
Trauma; other injuries (CC 166–168, 170–174)	19,691 (2.6%)	1914 (1.9%)	844 (3.1%)	541 (2.4%)	307 (2.6%)	500 (2.2%)	200 (1.6%)	1023 (3.0%)	1663 (2.8%)	2580 (2.3%)	1701 (2.1%)	397 (1.7%)	1162 (1.8%)	4916 (4.2%)	558 (2.7%)	287 (3.7%)	922 (2.3%)	176 (2.8%)	<0.001
History of COVID-19	8321 (1.1%)	955 (0.9%)	434 (1.6%)	272 (1.2%)	94 (0.8%)	255 (1.1%)	84 (0.7%)	466 (1.4%)	920 (1.6%)	1237 (1.1%)	629 (0.8%)	238 (1.0%)	809 (1.3%)	1564 (1.3%)	146 (0.7%)	75 (1.0%)	54 (0.1%)	89 (1.4%)	<0.001
Crude Mortality Rate	86,426 (11.3%)	15135 (14.7%)	2984 (11.1%)	2595 (11.4%)	1040 (8.7%)	2640 (11.5%)	1359 (11.0%)	4332 (12.9%)	7155 (12.1%)	11486 (10.3%)	9182 (11.1%)	3004 (12.8%)	7762 (12.2%)	10608 (9.1%)	2099 (10.1%)	1087 (14.2%)	3300 (8.4%)	658 (10.4%)	<0.001

CC, condition categories (Ref. 12).

### Hospitalisation rate and in-hospital mortality

**Fig. 1:**
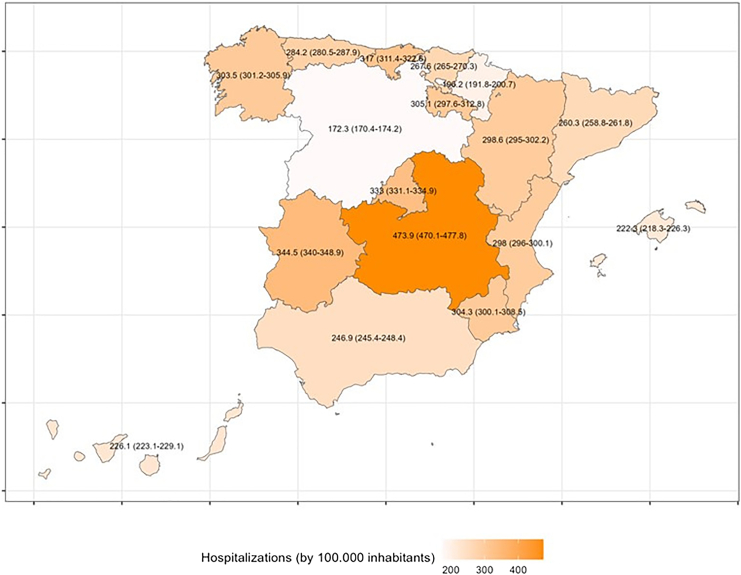
Age–sex–standardised hospitalisation for heart failure by autonomous community, Spain, 2016–22 (per 100,000 inhabitants).

The national age–sex–standardised hospitalisation rate was 255 per 100,000 inhabitants (crude 282 per 100,000). Marked variation across autonomous communities persisted after standardisation (Fig. 1 and Supplementary Material, Supplementary Table S5). The crude in-hospital mortality rate was 11.3% (86,426 episodes), with significant variation between autonomous communities (Table 1).

**Fig. 2:**
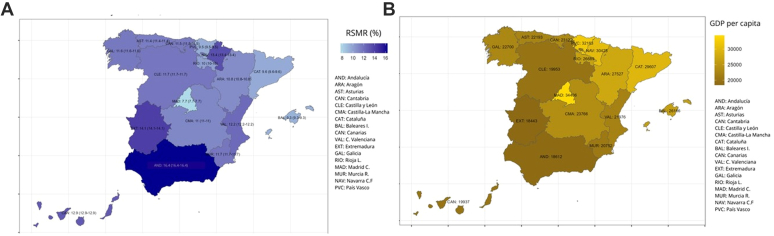
Risk-standardised in-hospital mortality rate (RSMR) and regional GDP per capita by autonomous community, Spain, 2016–22. (A) Risk-standardised in-hospital mortality (%) by autonomous community. (B) Per capita income (GDP per capita €).

The risk-adjustment models for mortality showed acceptable discrimination (AUROC: 0.71; CI 95%: 0.70–0.72) and calibration (Supplementary Material, Supplementary Figures S2 and S3). There was no evidence of collinearity among variables in the multilevel logistic regression model. The presence of cardiogenic shock at admission was the strongest predictor of in-hospital mortality (OR: 21.9; 95% CI: 20.2–23.7) (Supplementary Material, Supplementary Table S4). No statistically significant association was found between the hospitalisation rate and in hospital risk-standardised mortality rate due to autonomous community (r′ = −0.09; p = 0.74). Relevant differences were observed in the risk-standardised mortality rate between autonomous communities, with the highest risk-standardised mortality rate being more than double the lowest (16.4% in Andalucía vs. 7.7% in Madrid; Median Odds Ratio: 1.22) (Table 2 and Fig. 2A).

**Table 2:** Crude and risk-adjusted in-hospital mortality rates by Spanish autonomous community, 2016–2022.

Spanish autonomous communities	Number of episodes	CMR (%)	RSMR (%)	95% CI	
Andalucía	102,700	14.7	16.4	16.4	16.4
Aragón	26,970	11.1	10.8	10.8	10.8
Asturias	22,725	11.4	11.4	11.4	11.4
Baleares I	11,913	8.7	9.3	9.3	9.3
Canarias	22,937	11.5	12.9	12.9	13.0
Cantabria	12,313	11.0	11.5	11.5	11.5
Castilla y León	33,668	12.9	11.1	11.1	11.1
Castilla-La Mancha	58,940	12.1	11.7	11.7	11.7
Cataluña	111,603	10.3	9.6	9.6	9.6
C. Valenciana	82,378	11.1	12.2	12.2	12.2
Extremadura	23,423	12.8	14.1	14.1	14.1
Galicia	63,666	12.2	11.6	11.6	11.6
Madrid C.	116,703	9.1	7.7	7.7	7.7
Murcia R.	20,792	10.1	11.7	11.7	11.7
Navarra C.F.	7676	14.2	13.4	13.4	13.4
País Vasco	39,351	8.4	9.5	9.5	9.5
Rioja L	6325	10.4	10.0	10.0	10.0
Total	764,083	11.3			

CMR, crude mortality rate; RSMR, risk-standardised mortality rate; CI, confidence interval.

### Differences in hospital complexity between autonomous communities and their association with mortality

Notable differences were observed between autonomous communities in terms of the complexity of the hospitals where patients with HF were admitted (Table 3). Overall, 368,002 (48.2%) of patients were admitted to high-complexity hospitals (types 3 or 4 according to the RECALCAR classification), with communities ranging from 25.2% to 80.9%. A total of 256,830 (33.6%) were admitted to hospitals with a coronary care unit (range: 0.0%–77.2%), and 173,768 (22.7%) were admitted to hospitals with a cardiac intensive care unit (range: 0%–70.9%). The k-means clustering algorithm identified centres with more than 470 discharges per year as high-volume centres. In total, 528,628 (69.2%) of patients were admitted to high-volume hospitals (range 39.1%–87%). No statistically significant correlation was identified between differences in hospital complexity variables and risk-standardised mortality rate across autonomous communities.

**Table 3:** Structural indicators of hospital care for heart failure by autonomous community.

Autonomous community	Total	Andalucía	Aragón	Asturias	Baleares I	Canarias	Cantabria	Castilla y León	Castilla-La Mancha	Cataluña	C. Valenciana	Extremadura	Galicia	Madrid C.	Murcia R.	Navarra C.F.	País Vasco	Rioja L	p
**% Admission to**																			
Type 4	262,973	41,265	6524	3624	4535	8852	5936	20,938	5137	28,470	17,426	5652	27,622	59,972	3589	5446	12,867	5118	
	34.40%	40.20%	24.20%	15.90%	38.10%	38.60%	48.20%	35.50%	15.30%	25.50%	21.20%	24.10%	43.40%	51.40%	17.30%	70.90%	32.70%	80.90%	<0.001
Type 3 and 4	368,002	75,743	11,570	7730	4535	17,394	5936	26,699	15,015	37,354	20,747	7545	34,368	59,972	7135	5446	25,695	5118	
	48.20%	73.80%	42.90%	34.00%	38.10%	75.80%	48.20%	45.30%	44.60%	33.50%	25.20%	32.20%	54.00%	51.40%	34.30%	70.90%	65.30%	80.90%	<0.001
CU	256,830	26,597	6524	8180	4535	5250	5936	20,938	10,129	38,981	3515	5652	17,458	63,718	3589	5446	30,382	0	
	33.60%	25.90%	24.20%	36.00%	38.10%	22.90%	48.20%	35.50%	30.10%	34.90%	4.30%	24.10%	27.40%	54.60%	17.30%	70.90%	77.20%	0.00%	<0.001
CICU	173,768	7323	0	3624	0	4421	5936	20,938	9466	38,981	3515	5652	7180	48,419	0	5446	12,867	0	
	22.70%	7.10%	0.00%	15.90%	0.00%	19.30%	48.20%	35.50%	28.10%	34.90%	4.30%	24.10%	11.30%	41.50%	0.00%	70.90%	32.70%	0.00%	<0.001
High volume	528,628	70,452	11,651	12,431	4658	16,996	10,183	50,629	18,618	54,797	58,488	13,609	55,389	98,031	11,706	5481	30,497	5136	
	69.2%	68.6%	43.2%	54.7%	39.1%	74.1%	82.7%	85.9%	55.3%	49.1%	71%	58.1%	87%	84%	56.3%	71.4%	77.5%	81.2%	<0.001

U, coronary unit; CICU, cardiac intensive care unit. High-volume: ≥470 heart failure discharges per year.

### Association between health determinants and in-hospital mortality

Table 4 shows the raw values of the health determinants for each autonomous community used in our analyses.

**Table 4:** Health determinants by autonomous communities.

Autonomous communities	GDP pc	Normalised GDP pc	Average disposable income	Population at risk of poverty	Inequality (S80/S20 ratio)	Educational attainment	Unemployment rate	Smoking population	Risky alcohol consumption	Obesity	PM 10	PM 2.5
Andalucía	18,966	1.01	14,227	31.37	6.40	52.33	23.12	20.77	1.48	20.34	26.07	12.94
Aragón	28,031	1.49	18,498	15.41	4.67	65.80	11.24	19.33	1.21	15.74	16.09	10.32
Asturias	22,691	1.20	17,909	17.63	6.03	67.00	14.02	20.11	1.65	20.40	23.48	10.45
Baleares I	26,657	1.41	18,344	16.11	5.60	60.09	12.95	20.28	0.61	15.92	20.03	12.64
Canarias	20,275	1.08	14,305	30.54	7.07	57.94	21.97	19.96	0.43	18.38	27.66	9.30
Cantabria	23,557	1.25	17,577	16.90	5.03	67.54	11.77	19.79	0.52	15.59	20.71	9.35
Castilla y León	24,226	1.28	17,432	16.13	4.80	61.46	12.46	19.96	3.52	15.02	16.09	8.70
Castilla-La Mancha	20,327	1.08	14,725	27.79	5.74	51.51	18.10	21.22	1.16	17.36	25.86	10.31
Cataluña	30,027	1.59	20,152	14.53	5.57	63.13	12.28	20.51	1.29	14.57	23.56	14.22
C. Valenciana	22,333	1.18	15,573	24.53	5.97	59.47	16.29	20.28	1.41	17.71	18.40	11.14
Extremadura	18,858	1.00	13,146	33.14	5.01	53.56	22.60	23.17	1.71	16.05	13.43	6.77
Galicia	23,158	1.23	16,617	19.54	5.26	60.44	13.22	16.85	0.73	17.42	20.34	11.50
Madrid C.	34,981	1.86	20,780	15.94	6.50	73.76	12.47	17.28	1.41	14.65	19.27	10.37
Murcia R.	21,101	1.12	14,239	27.76	5.33	52.70	16.25	21.23	0.44	17.60	27.78	13.41
Navarra C.F.	30,909	1.64	21,324	9.21	4.57	69.97	10.21	17.75	2.81	14.30	14.12	10.63
País Vasco	32,687	1.73	22,316	10.24	5.04	73.36	10.25	16.56	2.21	13.68	16.49	9.51
Rioja L	27,102	1.44	18,451	14.03	4.91	62.43	11.12	18.98	0.80	15.38	20.29	9.53

GDP pc, average regional GDP during the period, in € per capita; PM 10, particulate matter <10 μm; PM 2.5, particulate matter <2.5 μm.

Among the selected health determinants, the following were excluded because of a high degree of correlation with the remaining determinants (R ≥ 0.5): average disposable household income, population at risk of poverty, level of education in the adult population, unemployment rate, percentage of the population who smoke, and obesity.

**Fig. 3:**
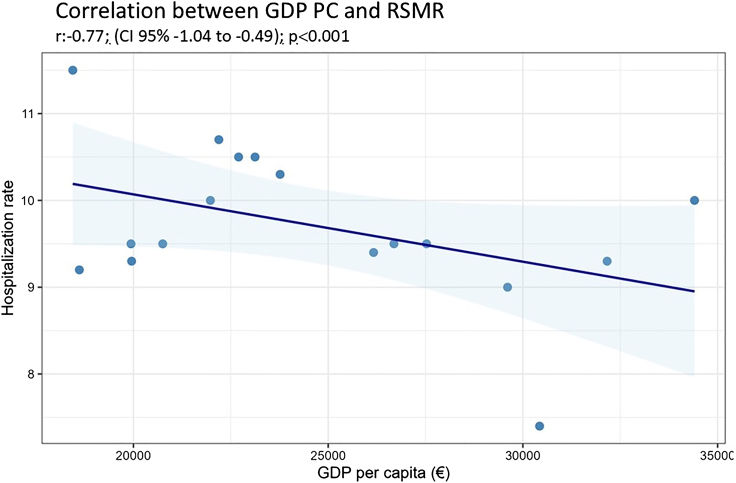
Correlation by autonomous community (AC) between per capita income (GDP p.c.) and risk-standardised in-hospital mortality (RSMR), Spain, 2016–22.

Linear regression analysis exploring the association between health determinants and risk-standardised mortality rate revealed a statistically significant association between adjusted GDP p.c. and risk-standardised mortality rate (βGDPpc = −0.77; 95% CI: −1.04 to −0.49; p < 0.001; R2 = 0.77) (Fig. 3). Statistically significant associations were observed for the percentage of admissions to type 3 and 4 hospitals and PM 2.5 particles, and non-significant associations for PM 10, all of which were clinically insignificant (regression coefficients <0.05) (Supplementary Material, Supplementary Table S6). A high degree of correlation was confirmed between regional GDP p.c. and the excluded health determinants: average disposable household income (R = 0.95), risk of poverty (R = −0.85), level of education in the adult population (R = 0.85), unemployment rate (R = −0.78), smoking rate (R = −0.71), and obesity rate (R = −0.75).

In the regression analysis including GDP p.c. stratified by income quartiles, autonomous communities in the higher quartiles presented a lower risk of in-hospital mortality for HF, with a significant progressive reduction as the quartile increased (Table 5).

**Table 5:** Odds ratios for quartiles of per capita income by autonomous community for risk-adjusted in-hospital mortality.

Quartile	GDP p.c. (€)	OR	95% CI	
1	19,182 ± 801	1.00		
2	22,336 ± 387	0.77	0.75	0.78
3	27,476 ± 2494	0.64	0.63	0.66
4	33,680 ± 1194	0.50	0.48	0.51

GDP p.c., Gross Domestic Product per capita (advance estimate, National Statistics Institute, 2022; mean ± standard deviation); OR, odds ratio; 95% CI, 95% confidence interval; €, euros.

## Discussion

Interregional inequalities in clinical outcomes in HF have been the subject of growing interest in the international literature.^5^^,^^6^^,^^15^, ^16^, ^17^, ^18^, ^19^, ^20^ However, studies quantifying this variability within public and decentralised health care systems, such as the Spanish system, which uses population-based databases and clinical risk adjustment methodologies, remain scarce. Furthermore, only a limitednumber of studies have systematically addressed the role of health determinants as potential explanatory factors for these disparities, particularly those beyond purely socioeconomic factors, such as social, environmental, or lifestyle-related factors.^21^^,^^22^ Our work, which is based on more than 760,000 HF hospital episodes between 2016 and 2022, represents one of the largest and most up-to-date analyses conducted in Europe on this issue.

Our main findings are as follows: first, we identified significant differences between autonomous communities in terms of hospitalisation rates and risk-standardised mortality rate among patients admitted for HF in Spain. Second, these differences were not associated with characteristics of hospital structure, such as care volume, as has been reported in previous studies conducted in Spain.^23^ In contrast, GDP p.c. was the only determinant showing a significant association with risk-standardised mortality rate, with higher-income regions exhibiting lower adjusted mortality. The association remained after accounting for year effects, with a small uptick in 2020 that likely reflects the post-pandemic care context and did not alter our main conclusion.

Our findings update and expand upon the results of a previous national study conducted in Spain during the period 2003–2015, which reported significant differences between autonomous communities in both hospitalisation rates and risk-standardised mortality rate in patients with HF. This study highlighted the need to explore the underlying factors driving such disparities, such as socioeconomic, health care, and organisational factors.^24^

Although the present study does not allow us to identify the specific mechanisms underlying the association between GDP p.c. and regional risk-standardised mortality rate, it is plausible that patients in regions with higher GDP p.c. benefit from stronger primary care and secondary prevention, greater access to medications and cardiac rehabilitation, more robust health education, healthier behaviours, and more supportive social services and living environments (including housing). These factors could facilitate earlier diagnosis and treatment of HF, reduce clinical severity at admission, and improve adherence to treatments and lifestyle modifications.

The differences in health outcomes between regions observed in our study are consistent with those reported in international research.^5^^,^^6^^,^^19^^,^^25^^,^^26^ In the United States, Liu et al. reported a greater burden of vascular risk factors in rural areas associated with social determinants such as poverty, low educational attainment, and food insecurity.^15^ Similarly, the European Society of Cardiology registry reported better risk-adjusted outcomes in northern (higher-income) countries than in southern countries and concluded that these differences might reflect variations in HF severity, inequalities in clinical management, available resources, and the structure of health care systems.^18^ In the Asian registry, lower-income countries exhibited worse outcomes.^19^ Our study, which focused on smaller and culturally more homogeneous regions than those of the countries included in these previous studies and was conducted within a publicly funded universal health care system, revealed significant inequalities between autonomous communities in risk-standardised mortality rate that are not explained by structural differences across hospitals. These findings suggest that, beyond individual risk and access to health care resources, regional socioeconomic disparities may play a relevant role in regional differences in cardiovascular care outcomes.^27^, ^28^, ^29^

Beyond HF, the accumulated body of evidence indicates that higher GDP per capita is associated with lower mortality and better outcomes across cardiovascular conditions, including acute myocardial infarction and stroke.^30^, ^31^, ^32^ Although ecological analyses do not allow individual-level causal inference, the convergence of findings across diverse settings supports the relevance of GDP as a marker of the health-system and social context associated with mortality.

No significant associations were observed for the other analysed social determinants, such as income inequality, the prevalence of excessive alcohol consumption, or levels of environmental pollution. The strong correlation between GDP p.c. and other key social indicators—such as average disposable income, educational attainment, unemployment, and the prevalence of smoking and obesity—suggests that GDP p.c. functions as a multidimensional, area-level proxy of the regional socioeconomic environment, encompassing material resources, education, employment, health behaviours, and elements of health-system capacity and access. In the absence of individual-level data, this measure provides an approximation of the aggregate impact of social determinants on hospital clinical outcomes; however, with the CMBD database, it was not possible to assess potential differences in the individual clinical management of patients.

From a public health perspective, these findings reinforce the need to consider the territorial context and socioeconomic dimensions when designing equitable health care policies. They also provide useful evidence for other countries with decentralised health care systems, where regional disparities may be shaped not only by structural differences within the health care system but also by the social conditions of the environments in which patients live and become ill.

This study has several limitations inherent to the use of administrative data, notably the lack of information on relevant clinical aspects such as the use, dosing/titration, and adherence to guideline-directed medical therapy and device therapy. In addition, multi-year regional measures of primary care access were unavailable. Our in-hospital mortality outcome does not include deaths occurring after discharge. Moreover, although the validity and concordance of key variables for risk adjustment are acceptable when the CMBD database has been compared with a clinical registry of hospital admissions for HF, they are not as robust as when the CMBD database is used for outcome analysis in acute coronary syndrome patients.^33^ Finally, it should be noted that the associations and correlations that were identified in this study are ecological in nature, and no causal inferences can be drawn between the analysed variables and the outcomes.

### Conclusions

We found interregional differences in hospitalisation rates and in-hospital mortality among patients admitted for HF in Spanish hospitals. These differences cannot be explained solely by age, comorbidities, or structural hospital differences between autonomous communities. In contrast, inequalities in GDP p.c. between regions were associated with differences in regional risk-standardised mortality rate. The disparities identified highlight the need for specific studies to further investigate the reasons behind these territorial differences and underscore the importance of incorporating social determinants at the individual level in health outcome research,^34^^,^^35^ whether comparing regions within the same country or between countries sharing similar sociopolitical and economic environments, such as the European Union.

## Contributors

All authors contributed to the conception and design of the study. NR, SF, NP, JLB, CP, and FJE contributed to data collection, curation, and analysis. CO, CB, LF, JMG, AC, LF, FJE, and NR contributed to the drafting and writing of the original manuscript. NR, SF, NP, JLB, CP, and FJE had access to the raw data and verified the underlying data. The corresponding author (CO) had full access to all the data and had final responsibility for the decision to submit for publication. All authors contributed to the interpretation of data, critically revised the manuscript for important intellectual content, and approved the final version for submission.

## Data sharing statement

The hospital discharge data used in this study are available upon request from the Spanish Ministry of Health through its Minimum Basic Data Set (CMBD), subject to institutional approval. Regional health and socioeconomic indicators were obtained from publicly available open-access sources, as detailed in the references.

## Editor note

The Lancet Group takes a neutral position with respect to territorial claims in published maps and institutional affiliations.

## Declaration of interests

All authors have completed the ICMJE disclosure forms and declare no competing interests.

## Appendix ASupplementary data

Supplementary Material

Abstract Spanish
