# Supplementary material for: Regional inequalities in heart failure hospitalisation and in-hospital mortality in Spain (2016–2022): a nationwide, observational study
Source: Lancet Reg Health Eur. 2025 Dec 2;61:101549. doi: 10.1016/j.lanepe.2025.101549 (PMC12720035; doi:10.1016/j.lanepe.2025.101549)

**Regional variations in hospitalisation and in-hospital mortality for heart failure in Spain: the impact of health determinants**

**Supplementary Material**

**International Classification of Diseases 10th Edition (ICD-10) Codes to Identify Heart Failure**

I11.0, I13.0, I13.2, I50.1, I50.20, I50.21, I50.22, I50.23, I50.30, I50.31, I50.32, I50.33, I50.40, I50.41, I50.42, I50.43, I50.810, I50.811, I50.812, I50.813, I50.814, I50.82, I50.83, I50.84, I50.89, I50.9

**Criteria for the Availability of Cardiac Intensive Care Unit (CICU) and Coronary Care Unit (CCU)**

Availability of CICU was considered when cardiology services treated critically ill cardiovascular patients, including those requiring invasive mechanical ventilation (1). CCU availability was considered when cardiology services treated critically ill cardiovascular patients but had to transfer those requiring invasive mechanical ventilation. Data on the availability of CICU and CCU were obtained from the survey conducted by the Ischemic Heart Disease and Acute Cardiovascular Care Association of the Spanish Society of Cardiology (2).

**References**
(1) Valle V, Alonso A, Arós F, Gutiérrez J, Sanz G. Clinical practice guidelines of the Spanish Society of Cardiology on requirements and equipment for coronary care units. Rev Esp Cardiol 2001; 54: 617-623.
(2) Worner F, San Román A, Sánchez PL, Viana Tejedor A, González-Juanatey JR. Care for patients with acute and critical cardiac diseases. Position statement of the Spanish Society of Cardiology. Rev Esp Cardiol. 2016;69:239–242.

***Table S1. RECALCAR classification of hospital complexity levels***

| **Type** | **Characteristics** |
| --- | --- |
| Type 1 | Cardiology departments or units without a catheterization laboratory. |
| Type 2 | Cardiology departments with a catheterization laboratory, no cardiovascular surgery service, and <400 PCI procedures/year. |
| Type 3 | Cardiology departments with a catheterization laboratory, no cardiovascular surgery service, and ≥400 PCI procedures/year. |
| Type 4 | Cardiology departments with a catheterization laboratory and an in-house cardiovascular surgery service. |
| PCI: percutaneous coronary intervention | |

Ref: Íñiguez Romo A, Bertomeu Martínez V, Rodríguez Padial L, Anguita Sánchez M, Ruiz Mateas F, Hidalgo Urbano R, Bernal Sobrino JL, Fernández Pérez C, Macaya de Miguel C, Elola Somoza FJ. The RECALCAR Project. Healthcare in the Cardiology Units of the Spanish National Health System, 2011 to 2014. Rev Esp Cardiol (Engl Ed). 2017 Jul;70(7):567-575

***Table S2. Health determinants by Autonomous Communities: indicators, definitions, and data availability (2016–2022)***

| **Indicator** | **Definition** | **Source** | **Availability** |
| --- | --- | --- | --- |
| **GDP per capita** | Average regional GDP during the period, in € per capita | Regional Accounts of Spain - Statistical Review 2019. GDP per capita. Advance estimate for 2022 | Entire period (2016-2022) (2021: provisional; 2022: advance) |
| **Normalized GDP per capita** | Regional GDP per capita (€) divided by the GDP of the Autonomous Community with the lowest GDP per capita | Regional Accounts of Spain - Statistical Review 2019. GDP per capita. Advance estimate for 2022 | Entire period (2016-2022) (2021: provisional; 2022: advance) |
| **Average disposable income** | Total income received by the household during the specified reference period, after deduction of income tax, wealth tax, and social security contributions, including received transfers | Living Conditions Survey. National Statistics Institute (INE) | Entire period (2016-2022) |
| **Population at risk of poverty** | Percentage of people whose annual income per consumption unit in the year prior to the survey is below the poverty risk threshold (set at 60% of the median income per consumption unit at national level) | Living Conditions Survey. National Statistics Institute (INE) | Entire period (2016-2022) |
| **Inequality (S80/S20 ratio)** | Ratio between the average income of the highest 20% of the population (highest quintile) and the average income of the lowest 20% (lowest quintile) | Living Conditions Survey. National Statistics Institute (INE) | Entire period (2016-2022) |
| **Educational attainment** | Percentage of the population aged 25-65 who have completed levels 3-8 (upper secondary education or higher) | Labour Force Survey. National Statistics Institute (INE) | Entire period (2016-2022) |
| **Unemployment rate** | Ratio between the unemployed population and the active population. Unemployed individuals are those aged 16 or over who, during the reference week: without work, available for work, and actively seeking employment. | Labour Force Survey. National Statistics Institute (INE) | Entire period (2016-2022) |
| **Smoking population** | % of daily smokers in the adult population. In 2011-2012, 2014, 2017, and 2020: aged 15 or over; in 2022: aged 16 or over | European Health Survey 2020, National Health Survey 2017. Ministry of Health - INE. Living Conditions Survey 2022, INE | 2017; 2020; 2022 |
| **Risky alcohol consumption** | % of individuals with risky alcohol consumption (weighted results, general population) | European Health Survey 2020, National Health Survey 2017. Ministry of Health - INE. Living Conditions Survey 2022, INE | 2017; 2020 |
| **Obesity** | Obesity prevalence (%) in the population aged 18 and over | European Health Survey 2020, National Health Survey 2017. Ministry of Health - INE. Living Conditions Survey 2022, INE | 2017; 2020 |
| **Air pollution PM10** | Population-weighted annual average concentration of PM10 (µg/m³) | Ministry for Ecological Transition and Demographic Challenge | Entire period (2016-2022) |
| **Air pollution PM2.5** | Population-weighted annual average concentration of PM2.5 (µg/m³) | Ministry for Ecological Transition and Demographic Challenge | Entire period (2016-2022) |
| **Abbreviations:** GDP: Gross Domestic Product; INE: National Statistics Institute; PM10: Particulate matter <10 μm; PM2.5: Particulate matter <2.5 μm; MSCBS: Ministry of Health, Consumer Affairs and Social Welfare. | | | |

***Table S3. Variables included in the risk adjustment model for in-hospital mortality in heart failure***

| **Variable** | **POA^1^** |
| --- | --- |
| Age (continuous variable from 18 years onward) |  |
| Sex (male) |  |
| Year (categoric) |  |
| History of coronary artery bypass grafting |  |
| History of percutaneous coronary intervention |  |
| Metastatic cancer, acute leukemia, and other severe cancers (CC 8-9) |  |
| Diabetes mellitus (DM) complications, except proliferative retinopathy (CC 17-19, 123) | CC17 (Acute complications of DM) |
| Protein-calorie malnutrition (CC 21) |  |
| Chronic liver disease (CC 27-29) |  |
| Dementia or other brain disorders (CC 51-53) |  |
| Severe psychiatric disorders (CC 57-59) |  |
| Hemiplegia, paraplegia, paralysis, functional disability (CC 70-74, 103-104, 189-190) | CC 103 (Hemiplegia; hemiparesis); CC 104 (Monoplegia; other paralytic syndromes) |
| Cardiogenic shock (ICD-10 code: R57.0) | Yes |
| Acute pulmonary edema and other severe complications of heart failure (CC 84), excluding cardiogenic shock | Yes |
| Acute myocardial infarction (CC 86) | Yes |
| Unstable angina / other acute ischemic heart diseases (CC 87) | Yes |
| Coronary atherosclerosis or angina (CC 88-89) |  |
| Rheumatic and valvular heart disease (CC 91) |  |
| Hypertension (CC 95) |  |
| Stroke (CC 99-100) | CC 99 (Intracranial hemorrhage); CC 100 (Ischemic or unspecified stroke) |
| Vascular disease and complications (CC 106-108) | CC 106 (Peripheral arteriosclerosis with ulceration or gangrene); CC 107 (Vascular disease with complications); CC 108 (Vascular disease) |
| COPD (Chronic Obstructive Pulmonary Disease) (CC 111) |  |
| Pneumonia (CC 114-116) | CC 114 (Aspiration and bacterial pneumonia); CC 115 (Pneumococcal pneumonia; aspiration; empyema) |
| Renal failure (CC 135-140) | CC 135 (Acute renal failure); CC 140 (Unspecified renal failure) |
| Trauma; other injuries (CC 166-168, 170-174) | CC 166-168 (Severe head injury; major trauma; unspecified contusion); CC 170 (Hip fracture/dislocation); CC 171 (Major fracture except skull, vertebrae, or hip); CC 173 (Traumatic amputation and complications); CC 189 (Lower limb amputation status/complications); CC 190 (Lower limb amputation status/complications) |
| History of COVID-19 |  |
| POA: Present on admission; CC: Condition Categories (comorbidity groupings); ICD-10: International Classification of Diseases, 10th Revision; DM: Diabetes Mellitus; COPD: Chronic Obstructive Pulmonary Disease.  ^1^ Whan specified must be registered as POA to be considered a comorbidity present on admission. | |

***Table S4. Risk-adjustment model for in-hospital mortality for heart failure. Predictive variables***

|  | Odds ratio | | 95% conf. interval | | P | |
| --- | --- | --- | --- | --- | --- | --- |
|  |  |  | |  | |  |
| Sex (man) | 0,933 | 0,918 | | 0,949 | | <0,001 |
| Age (continuous variable) | 1,053 | 1,052 | | 1,054 | | <0,001 |
| Year (2016) |  |  | |  | |  |
| 2017 | 0,987 | 0,960 | | 1,015 | | 0,373 |
| 2018 | 0,975 | 0,948 | | 1,003 | | 0,077 |
| 2019 | 0,962 | 0,935 | | 0,989 | | 0,006 |
| 2020 | 1,041 | 1,011 | | 1,071 | | 0,006 |
| 2021 | 0,943 | 0,917 | | 0,970 | | <0,001 |
| 2022 | 0,925 | 0,899 | | 0,953 | | <0,001 |
| History of percutaneous transluminal coronary angioplasty (PTCA) | 0,837 | 0,811 | | 0,864 | | <0,001 |
| History of coronary artery bypass graft (CABG) surgery | 0,979 | 0,932 | | 1,028 | | 0,401 |
| Metastatic cancer, acute leukemia and other severe cancers (CC 8-9) | 2,698 | 2,601 | | 2,799 | | <0,001 |
| Diabetes mellitus (DM) or DM complications except proliferative retinopathy (CC 17-19, 123) | 0,853 | 0,840 | | 0,866 | | <0,001 |
| Protein-calorie malnutrition (CC 21) | 1,453 | 1,392 | | 1,517 | | <0,001 |
| Chronic liver disease (CC 27-29) | 1,634 | 1,569 | | 1,702 | | <0,001 |
| Dementia or other specified brain disorders (CC 51-53) | 1,354 | 1,327 | | 1,382 | | <0,001 |
| Major psychiatric disorders (CC 57-59) | 1,110 | 1,025 | | 1,203 | | 0,011 |
| Hemiplegia, paraplegia, paralysis, functional disability (CC 70-74, 103-104, 189-190) | 1,358 | 1,256 | | 1,469 | | <0,001 |
| Cardiogenic shock (R57.0) | 21,879 | 20,220 | | 23,675 | | <0,001 |
| Cardio-respiratory failure and shock (CC 84), plus ICD-10-CM codes R09.01 and R09.02,  except cardiogenic shock | 1,650 | 1,625 | | 1,676 | | <0,001 |
| Acute myocardial infarction (CC 86) | 1,830 | 1,700 | | 1,971 | | <0,001 |
| Unstable angina and other acute ischemic heart disease (CC 87) | 1,602 | 1,494 | | 1,718 | | <0,001 |
| Coronary atherosclerosis or angina (CC 88-89) | 0,973 | 0,953 | | 0,994 | | 0,011 |
| Valvular and rheumatic heart disease (CC 91) | 0,903 | 0,889 | | 0,917 | | <0,001 |
| Hypertension (CC 95) | 0,962 | 0,942 | | 0,981 | | <0,001 |
| Stroke (CC 99-100) | 2,826 | 2,559 | | 3,120 | | <0,001 |
| Vascular disease and complications (CC 106-108) | 1,106 | 1,076 | | 1,136 | | <0,001 |
| Chronic obstructive pulmonary disease (COPD) (CC 111) | 0,938 | 0,918 | | 0,958 | | <0,001 |
| Pneumonia (CC 114-116) | 2,086 | 2,027 | | 2,147 | | <0,001 |
| Renal failure (CC 135-140) | 1,668 | 1,643 | | 1,695 | | <0,001 |
| Trauma; other injuries (CC 166-168, 170-174) | 1,307 | 1,255 | | 1,362 | | <0,001 |
| History of COVID-19 | 1,213 | 1,136 | | 1,296 | | <0,001 |

***Table S5: Crude and age- and sex-adjusted hospitalisation rates for heart failure by autonomous community***

| **Autonomous Communities** | **Crude Hospitalisation Rate** | **Hospitalisation Rate**  **(adjusted for sex and age)** | **IC95%** | |
| --- | --- | --- | --- | --- |
| Andalucía | 214,0 | 246,9 | 245,4 | 248,4 |
| Aragón | 351,4 | 298,6 | 295,0 | 302,2 |
| Asturias | 366,3 | 284,2 | 280,5 | 287,9 |
| Islas Baleares | 173,4 | 222,3 | 218,3 | 226,3 |
| Canarias | 176,5 | 226,1 | 223,1 | 229,1 |
| Cantabria | 358,8 | 317,0 | 311,4 | 322,6 |
| Castilla y León | 233,7 | 172,3 | 170,4 | 174,2 |
| Castilla - La Mancha | 503,5 | 473,9 | 470,1 | 477,8 |
| Cataluña | 258,1 | 260,3 | 258,8 | 261,8 |
| Comunidad Valenciana | 286,1 | 298,0 | 296,0 | 300,1 |
| Extremadura | 376,7 | 344,5 | 340,0 | 348,9 |
| Galicia | 392,6 | 303,5 | 301,2 | 305,9 |
| Comunidad de Madrid | 306,8 | 333,0 | 331,1 | 334,9 |
| Murcia | 249,4 | 304,3 | 300,1 | 308,5 |
| Navarra | 206,8 | 196,2 | 191,8 | 200,7 |
| País Vasco | 310,0 | 267,6 | 265,0 | 270,3 |
| La Rioja | 347,8 | 305,1 | 297,6 | 312,8 |
| Total | 281,7 | 255,5 | 254,6 | 256,3 |

***Table S6. Linear Regression Analysis of the Association Between Health Determinants and Risk-Adjusted Mortality for Heart Failure, by Autonomous Community***

| **Risk-adjusted in-hospital mortality** | **Regression coefficient** | **95% CI** | **p-value** |
| --- | --- | --- | --- |
| Normalized GDP | -0,770 | -1.390 to -0.488 | <0.001 |
| Type 3 and 4 Hospitals | 0,005 | 0.002-0.009 | 0.008 |
| PM10 | -0.024 | -0.042 to - -0.05 | 0.017 |
| PM2.5 | 0.041 | 0.000 to 0.082 | 0.05 |
| Abbreviations: RAMER: Risk-Adjusted Mortality for Heart Failure by Autonomous Community; GDP: Gross Domestic Product; PM10: Particulate matter <10 μm; PM2.5: Particulate matter <2.5 μm. | | | |

***Figure S1. Exclusions flowchart***

***Figure S2. Discrimination and calibration of the risk adjustment model for in-hospital mortality in heart failure (2016–2022)***

| ***Discrimination*** | ***Calibration*** |
| --- | --- |
|  |  |

***Figure S3. Area under ROC curve for the hospitals included in the estudy***

***Figura S4: Correlation between GDP per capita and the age- and sex-standardised heart-failure hospitalisation rate by autonomous community.***


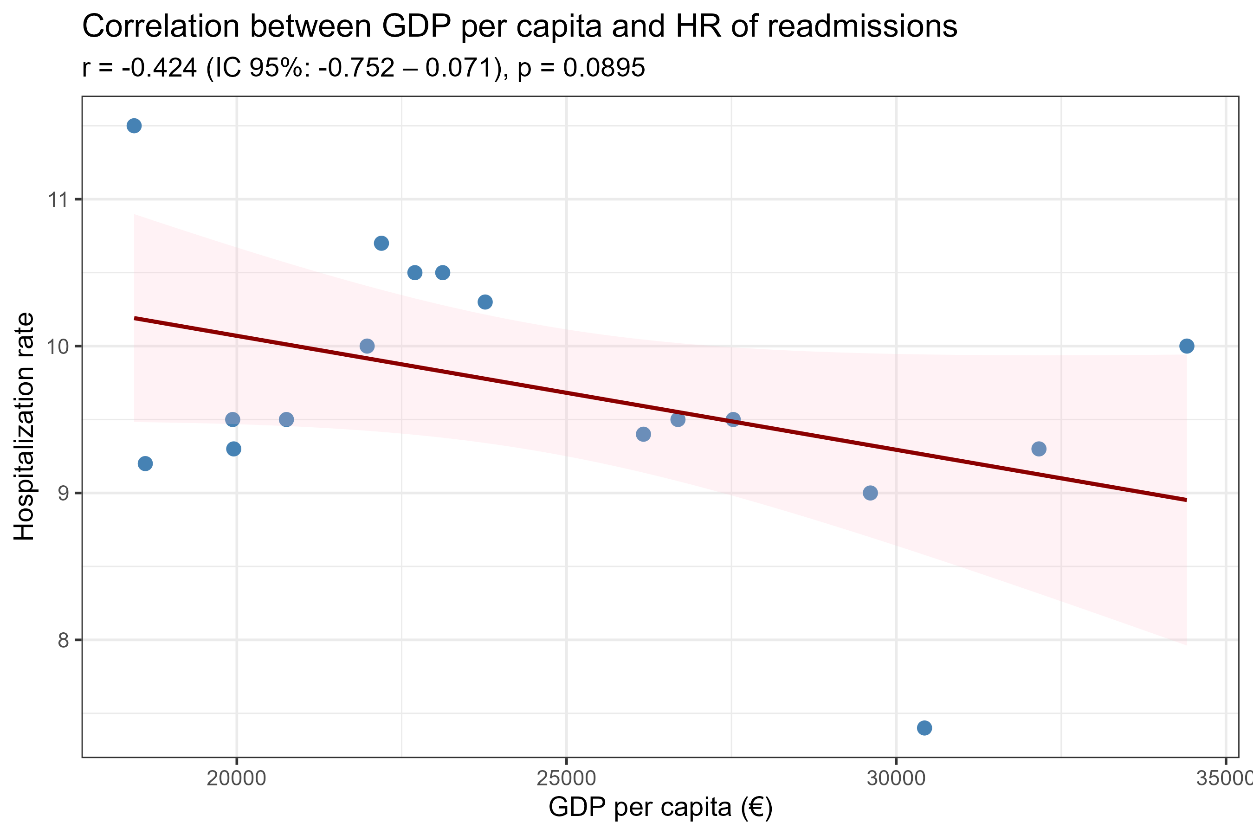

Supplement: Supplementary Material [file mmc1.docx]
