# Supplementary material for: Regional inequalities in heart failure hospitalisation and in-hospital mortality in Spain (2016–2022): a nationwide, observational study
Source: Lancet Reg Health Eur. 2025 Dec 2;61:101549. doi: 10.1016/j.lanepe.2025.101549 (PMC12720035; doi:10.1016/j.lanepe.2025.101549)
Supplement: Supplementary file 2 — Abstract Spanish [file mmc2.docx]

**Editor disclaimer:** This translation in Spanish was submitted by the authors and we reproduce it as supplied. It has not been peer reviewed. Our editorial processes have only been applied to the original abstract in English, which should serve as reference for this manuscript.

**Antecedentes:**
La insuficiencia cardiaca sigue siendo una de las principales causas de hospitalización y mortalidad en todo el mundo, con marcadas disparidades regionales en los resultados clínicos. Estas variaciones se han relacionado con diferencias en las condiciones socioeconómicas, los determinantes de salud y la organización del sistema sanitario. El objetivo de este estudio fue analizar las diferencias regionales en las tasas de hospitalización y la mortalidad intrahospitalaria por insuficiencia cardiaca entre las comunidades autónomas de España, y examinar su asociación con las características hospitalarias y los determinantes de salud regionales.

**Métodos:**
Realizamos un estudio nacional, retrospectivo y poblacional utilizando la base de altas hospitalarias de España (Conjunto Mínimo Básico de Datos, CMBD) para identificar a los adultos (≥18 años) dados de alta de hospitales generales públicos del Sistema Nacional de Salud (SNS) con diagnóstico principal de insuficiencia cardiaca, entre el 1 de enero de 2016 y el 31 de diciembre de 2022. Se analizaron las diferencias entre comunidades autónomas en las tasas de hospitalización y en la mortalidad intrahospitalaria estandarizada por riesgo, así como sus asociaciones con características hospitalarias y determinantes de salud regionales.

**Resultados:**
Se analizaron 764.083 hospitalizaciones por insuficiencia cardiaca. La edad media fue 80,8 ± 10,6 años. La tasa de hospitalización estandarizada por edad y sexo fue de 255 por 100.000 habitantes, con diferencias significativas entre comunidades autónomas. La tasa bruta de mortalidad intrahospitalaria fue 11,3 % (86.426 episodios), con una variación regional sustancial en la tasa de mortalidad intrahospitalaria estandarizada por riesgo (RSMR) (7,7–16,4 %). No se encontraron asociaciones entre las características hospitalarias y la RSMR a nivel regional. Los determinantes de salud regionales mostraron alta colinealidad; entre todos los evaluados, el producto interior bruto per cápita se asoció de forma independiente con una RSMR más baja (β_{PIBpc} = −0,77; IC 95 %: −1,04 a −0,49; p<0,001; R² = 0,77).

**Interpretación:**
Se observaron diferencias regionales significativas en las tasas de hospitalización y en la mortalidad intrahospitalaria estandarizada por riesgo en pacientes hospitalizados por insuficiencia cardiaca. Las disparidades en el PIB per cápita regional podrían explicar parcialmente estas diferencias. Estos resultados tienen implicaciones clínicas y de política sanitaria, al subrayar la necesidad de considerar los determinantes socioeconómicos al analizar resultados en salud y al diseñar políticas públicas dirigidas a reducir las desigualdades. Nuestros hallazgos pueden ser relevantes para otros países europeos con sistemas públicos de salud similares.
